# Principles and Practice of Physical Diagnosis

**Published:** 1912-04

**Authors:** 


**Principles and Practice of Physical Diagnosis. John C. Da Costa,** Jr., M.D., Philadelphia, W. B. Saunders Co., 1911. 557 pages, 225 original illustrations.

This is the second revised edition and is a most estimable book following the customary arrangement but with many allusions to pathology, illustrated from specimens, with brief references to X-rays and other more modern methods. The illustrations of methods of inspection, percussion, etc., follow to a certain degree the excellent innovation of Butler. Fig. 24—The thorax of incipient phthisis, represents a full breasted, broad waisted young woman with just about the ideal amount of subcutaneous fat. We don't question the diagnosis for we have occasionally found marked physical signs in chests apparently normal, but the inspection signs, if any, are not conspicuous. In mentioning auscultatory percussion, the author gets into deep water. This very valuable method might better be omitted altogether than discussed so briefly and with so many omissions. Tuning-fork auscultation is ascribed to Warder, without date. We have always supposed that we originated this method. At any rate the essay presented to the Medical Society of the State of New

York in 1894 and published with illustrations of tuning-fork, electric buzzer, stethoscopes, etc., in the 1895 Transactions and in the *Philadelphia Medical and Surgical Reporter* April 27 and June 1, 1895, was not questioned so far as priority was concerned. A. L. B.

